# Sorting Science From Marketing in the Era of Data-Driven Biological Aging Clocks

**DOI:** 10.2196/102951

**Published:** 2026-06-09

**Authors:** Jenna Congdon

**Keywords:** biological aging, biomarker, DNA methylation, photoplethysmography, heart rate variability, chronic obstructive pulmonary disease, wearable electronic devices

## Abstract

Consumer wearables provide users with a wealth of data, including an estimation of their “biological age.” In this *News and Perspectives* article, JMIR Correspondent Jenna Congdon reports on the accuracy and utility of age prediction by wearables.

**Key Takeaways:**

- Wearable “biological age” scores are based on proxy data, like sleep, heart rate, and activity. They offer trends and insights, not a definitive measure of health or aging.
- Aging clocks can support prevention and behavior change, especially around cardiovascular health and lifestyle habits, but interpreting results without clinical context may increase anxiety or misrepresent overall health.
- New US regulations easing oversight for some wearable health devices raise concerns about health data privacy.

You just turned 38, but the smartwatch you got for your birthday tells you that your “biological age” is 42! For the last several weeks, it’s been tracking your sleep, exercise, and heart rate. You can’t help but worry a bit—is this a real reflection of your health status?

The concept of an “aging clock” has been rapidly adopted by companies marketing biosensor-enabled wearable devices. These devices—most often watches or rings—measure markers, such as heart rate variability, sleep patterns, and exercise habits, to approximate the biological age of the wearer; in other words, they assess how that person’s data compare to a cohort of healthy individuals of a given age.

The medical field has likewise developed an array of aging clock models based on data from a number of biological markers. Regardless of how they’re measured, aging clocks are complex models, and the data they provide need both attention to context and careful interpretation to offer useful insights.

**Figure:**
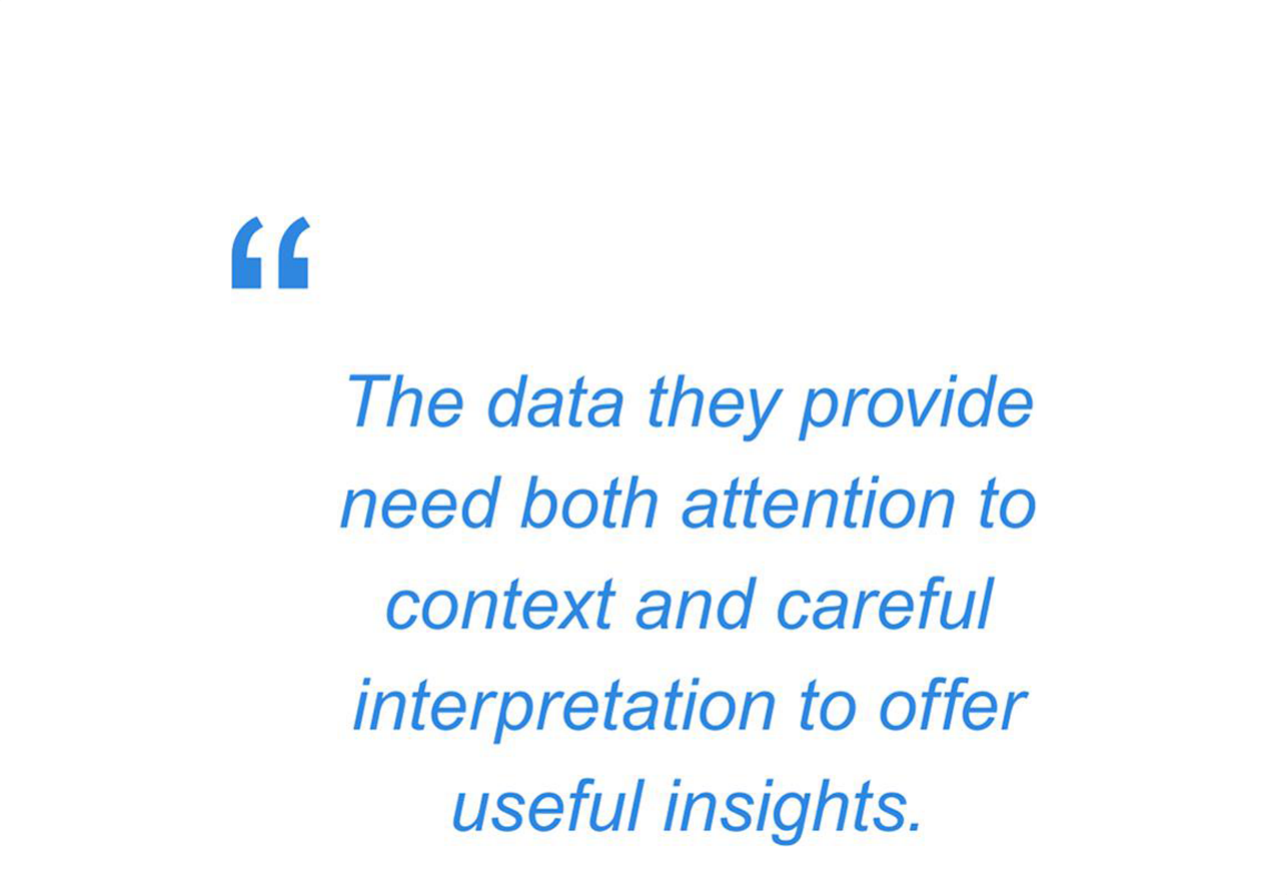


## The Science Behind Age Prediction

Aging clocks are biomarker-based predictive models that use molecular, cellular, and physiological data to estimate biological age. Though typically associated with a date on the calendar, aging is multifactorial and unique to each individual. One person may be frail at 75, while another runs marathons. Health care providers often instinctively assess a person’s perceived age alongside their calendar age to evaluate their overall clinical picture. Since chronological age is a leading risk factor for chronic disease and functional impairment, predicting age-based decline is a powerful prognostication tool that may offer the opportunity for more incisive and robust preventative health care.

Clinical aging clocks come in many forms. To name a few, epigenetic clocks use DNA methylation and inflammatory markers to estimate chronological age, phenotypic clocks use common clinical measures and blood work to estimate mortality, and proteomic clocks measure a large number of circulating plasma proteins to predict disease morbidity and mortality. These models were developed for the research setting, but they are slowly making their way into some health care providers’ prognostic repertoires as the demand for care for the aging population grows.

Some wearable devices use a different method of measuring age: PpgAge. This method uses photoplethysmography to collect cardiovascular and respiratory data passively, with a recent iteration—AI-PpgAge—using deep learning to help generate age estimates. In addition, these devices often track sleep and exercise patterns, as well as habitual activities, such as smoking. While each model does correlate with a different aspect of aging, consumer models risk the oversimplification of data and lack provider insight for assisting users in data interpretation.

## Can Wearables Accurately Estimate Biological Age?

Russell Bowler, MD, PhD, is the chair of the Department of Genome Sciences and Systems Biology at the Cleveland Clinic, where he specializes in proteomics and lung disease. He explains that “[a]n aging clock is really a statistical model that estimates where someone sits on the aging-related trajectory…it doesn’t really measure a true age in a literal sense. They typically capture multiple aspects of biology and behavior.”

He goes on to explain that aging clocks, in the clinical sense, are often organ-specific. “For instance, wearables might be measuring your cardiovascular fitness, and that’s not necessarily the same thing as your cardiovascular age. [It] doesn’t necessarily reflect, for instance, brain health and brain age.”

Interpretation of data from wearables requires context and an understanding that these metrics are not universally validated for the prediction of clinical outcomes. Still, an increase in PpgAge is associated with an increased incidence of cardiac events and metabolic disease, making it a convenient summary of a user’s physiology. Wearable users should be reminded that while these measurements may be insightful, they don’t offer a direct correlation with a person’s “true” age or health status.

## When Aging Becomes a Single Score

The primary issue with consumer-facing biological aging clocks is the reduction of complex biology into a single metric. While the aim of these devices is increased data accessibility, wearables risk a translation gap, which can lead to user anxiety or a misinterpretation of health status.

Wearable devices do offer benefits. For example, they provide real-time feedback on one’s activity levels, which may motivate increased movement throughout the day. The aggregate data from wearable devices can also become a predictor for chronic disease, particularly cardiovascular disease.

## How Aging Clocks Can Improve Real-World Health Outcomes

When applied clinically, scientifically modeled aging clocks can translate into improved health outcomes for patients. Bowler’s work on metabolic aging in lung disease provides an example. Bowler’s lab studies how advanced metabolic age on the cellular level correlates with the incidence of chronic obstructive pulmonary disease, which is a chronic lung disease typically seen in older people.

Smoking has been shown to decrease lung function, thus increasing one’s “lung age.” When smokers were told that their lung age was older than their chronologic age, they were more motivated to quit. Aging clocks may be used by clinicians as a talking point to help patients understand their own physiology and provide motivational feedback on lifestyle habits.

## New Wearables Guidelines in the United States

Recent changes to federal regulations in the United States impact how health data collected by wearable devices are protected and shared. In January 2026, the US Food and Drug Administration (FDA) loosened regulatory oversight around safety and data protection for many wearable health devices, including devices such as step counters, sleep trackers, and smartwatches. This opens consumers to possible data breaches of their personal health information, leading to concern over privacy and data sharing.

## What This Means for You and Your Wearable

Users of wearable health tracking devices should know that wearables do not directly measure the in-depth biomarkers used in scientifically validated aging clocks and are not definitive measures of aging. Instead, they use proxy data to generate aging-related insights. These data can be both motivating and insightful for the average wearer by increasing awareness of one’s lifestyle habits and providing a potential starting point for clinical conversations with health care providers.

Bowler recommends that wearable users consider the context of these numbers and “...let them help you motivate for change. Knowing that my biologic age is 10 years greater than my chronologic age doesn’t really help that much, but knowing that my cardiovascular fitness is 10 years more than my chronologic age might motivate me to exercise more.”

In short, wearables don’t measure the same complex biological markers as research-developed aging clocks do; it’s useful to think of them as different measuring tools for different purposes. Your watch isn’t *really* telling you you’re older than your age on the calendar, but it may be giving you a hint that some of your habits could use a health-conscious boost.

